# Multiple Effects of Resveratrol on Osteosarcoma Cell Lines

**DOI:** 10.3390/ph15030342

**Published:** 2022-03-11

**Authors:** Angela De Luca, Daniele Bellavia, Lavinia Raimondi, Valeria Carina, Viviana Costa, Milena Fini, Gianluca Giavaresi

**Affiliations:** IRCCS Istituto Ortopedico Rizzoli, CS Surgical Sciences and Technologies—SS Omics Science Platform for Personalized Orthopedics, 40136 Bologna, Italy; daniele.bellavia@ior.it (D.B.); lavinia.raimondi@ior.it (L.R.); valeria.carina@ior.it (V.C.); viviana.costa@ior.it (V.C.); milena.fini@ior.it (M.F.); gianluca.giavaresi@ior.it (G.G.)

**Keywords:** resveratrol, natural compound, proliferation, invasion, apoptosis, chemotherapeutic agents, doxorubicin, cisplatin, osteosarcoma

## Abstract

Osteosarcoma (OS) is the most common primary bone sarcoma affecting the life of pediatric patients. The clinical treatment faces numerous difficulties, including the adverse effects of chemotherapies, chemoresistance, and recurrences. In this study, the effects of resveratrol (RSV), a natural polyphenol, on OS cell lines were investigated to evaluate its action as an adjuvant therapy to the current chemotherapy regimens. RSV exhibited multiple tumor-suppressing activities on OS cell lines, inducing a series of critical events. We found (1) a cell growth inhibition due to an increase in cell distress, which was, in part, due to the involvement of the AKT and caspase-3 pathways, (2) an increase in cellular differentiation due to major gene expression levels of the osteoblastic differentiation genes, (3) an inhibition of IL-6 secretion due to an epigenetic effect on the IL-6 promoter, and (4) an inhibition of OS cells migration related to the decrease in IL-8 secretion levels due to an epigenetic effect on its promoter. Finally, the cotreatment of RSV with doxorubicin and cisplatin increased their cytotoxic effect on OS cells. Although further investigations are mandatory, it seems RSV might be a promising therapeutic adjuvant agent for OS cell treatment, exerting an antitumor effect when combined with chemotherapy.

## 1. Introduction

Osteosarcoma is the most common primary bone tumor in children and adolescents. Although the prognosis of OS has improved significantly with the improvement in treatment methods, the efficacy of treatment remains unsatisfactory. To date, the combination of surgery and associated chemotherapy has been the conventional therapeutic regimen for OS patients [1]. Chemotherapeutic agents, as well as methotrexate, cisplatin (CIS), doxorubicin (DOX), and ifosfamide, are used in a combined manner to treat OS with the goal of improving their actions, exploiting their different mechanisms of action targeting tumor cells at several levels [2]. Unfortunately, despite the diagnostic and therapeutic improvements, the percentage of patients with osteosarcoma who show minor benefits from these treatments has not yet been reduced [3]. The main causes for the poor prognosis of OS are recurrences and metastases, and the 5-year survival rate in patients after recurrence and lung metastasis is less than 20% [4]. Unfortunately, this discomforting picture is also often due to the failure of chemotherapy associated with chemo-resistance phenomena [5], besides many side effects of chemotherapeutic agents, such as cardiotoxicity, hepatotoxicity, and renal toxicity [6], leading to easy consequent cases of tumor recurrence and progression.

For these reasons, new strategies are needed today to improve the treatment of OS with new and effective agents. In recent years, particular attention has been paid to natural compounds [7]. Different plants are known as a rich source of natural products, each of which has different chemical structures and relevant pharmacological activities [8]. In fact, natural compounds have always been one of the most important and relevant assets in drug discovery and development [9], and they have been used by humans since the dawn of time for the maintenance and improvement of health and the prevention of diseases [10]. To date, several articles have shown the protective role of plant-derived bioactive compounds combined with the therapeutic agents to promote synergistic effects, reducing the side effects associated with the chemotherapeutic agent and/or enhancing the therapeutic efficacy [9].

Trans-3,4′,5-trihydroxystilbene, best known as resveratrol (RSV) is an edible polyphenolic phytoalexin found in several plants, including blueberries, mulberries, cranberries, peanuts, grapes, and wine [11,12]. A wide range of biological and pharmacological activities have been reported for RSV in various cell types and also in normal osteoblasts and osteoclasts in which it regulates cell proliferation, cellular senescence and apoptosis, and inflammation processes, reducing the activity of NF-κB and MAPK, and also acts through an epigenetic control, modulating the expression and activity of sirtuin-1 (SIRT-1), which is capable of increasing osteoblast survival and differentiation [13].

In pathological conditions, RSV exerts the anti-inflammatory, antioxidant effects [14] and anticancer activities via targeting oxidative systems, inducing cell-cycle arrest, apoptosis, or autophagy [15]. Indeed, many articles have reported the effects of RSV on various cancer models, highlighting its role in the suppression of the development and progression of cancer cells by the modulation of several cellular mechanisms, including apoptosis, cell cycle arrest, and the activation of transcription factors. [16]. A beneficial role of RSV was found in several kinds of tumor, as reported in the literature, including gastric [17], prostate [18], and breast cancers [19] and leukemia [20]. As reported by Xu et al., the RSV modulates the PTEN/Akt signaling pathway, inhibiting the epithelial–mesenchymal transition in gastric cancer, obtaining a reduction of chemoresistance as the final result [17]. Furthermore, Selvaraj et al. showed us that the downregulation of the STIM1 and mTOR pathway operated by RSV induces autophagic cell death in prostate cancer [18]. Meng et al. have reported how RSV treatment upregulated the expression of PTEN and reduced the expression of the PI3K/AKT pathway, suppressing proliferation and inducing apoptosis in leukemia cells [20]. Studies on the effect of RSV on OS confirmed a strong inhibition of cell growth [7,21,22], but the mechanism of action by which RSV works is not yet defined. 

The aims of the present study are to investigate:

- the inhibitory effects of RSV on different OS cell lines that best represent the various typical features of OS;
- the several mechanisms of action of RSV on gene and protein expression, on cell differentiation, motility, and cytokine secretion, the latter regulated by the epigenetic mechanism induced by RSV;
- the RSV synergistic effects with the chemotherapeutic agents during the cotreatment of OS cell lines. In particular, the last aspect is aimed at evaluating the possibility of reducing the concentration used in therapy with DOX and CIS to reduce the phenomena of chemoresistance and the various side effects that they induce on patients.

## 2. Results

### 2.1. Resveratrol Inhibits OS Cells Proliferation

**Figure 1:**
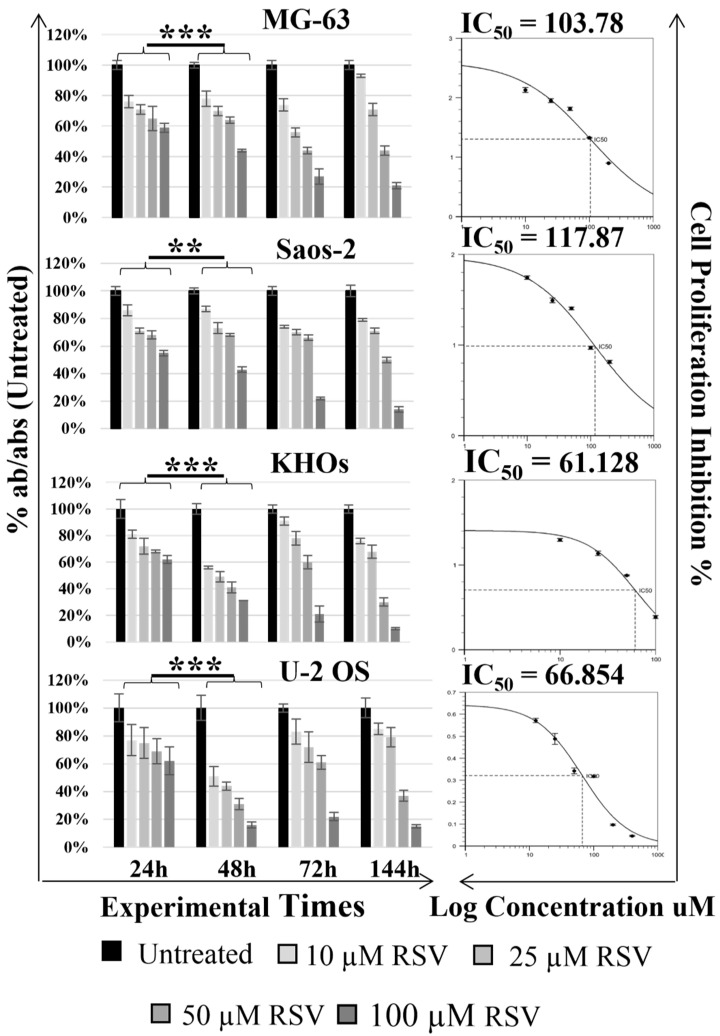
Effect of resveratrol on the cellular proliferation of OS cell lines. Cell viability was tested by WST-1 assay at various times after 48 h. As reported in each histogram, the absorbance values were converted into percentage absorbance values with respect to the untreated cells of each cell line. RSV treatment effects a significant reduction of cell proliferation after 48 h (** symbols correspond to *p* < 0.005 and *** symbols to *p* < 0.0005). All experiments were triplicated, with the data expressed as mean ± SD. On the right of each histogram are reported the graphs of the IC_50_ values of RSV calculated for each cell line after 48 h. The IC_50_ values were estimated by: https://www.aatbio.com/tools/ic50-calculator-v1, accessed on 1 February 2022.

The WST-1 assay was used to detect the effects of different concentrations of RSV (0, 10, 25, 50, and 100 µM) on different human OS cell lines (MG-63, Saos-2; KHOS, U-2 OS) at different experimental times (24, 48, 72, and 144 h). The results of the WST-1 assay showed that the proliferation of OS cells treated with RSV was significantly suppressed in a dose- and time-dependent manner (Figure 1).

In fact, a significant reduction in proliferation (*p*-value interval, [0.0005, 0.005]), especially after treatments at the highest concentrations of RSV (50–100 µM RSV), was observed at 48 h compared to 24 h in each cell line. Based on the data obtained from the 48 h WST-1 assay and using a free online program (https://www.aatbio.com/tools/ic50-calculator-v1 accessed on 14 February 2022), we were able to estimate the inhibitory concentration of 50% (IC_50_) of RSV at 48 h, which corresponds to 103.78 and 117.87 µM for the MG-63 and Saos-2 cell lines, respectively, and 61.13 and 66.85 µM for the KHOS and U-2 OS cell lines. Following these results, we set the experimental time at 48 h and the RSV concentration at 60 and 120 µM for all treatments because these include the IC_50_ interval calculated for OS cells. These experimental settings were also tested on not-tumorigenic cells such as NHOst and hMSC (Figure S1). WST-1 results showed no significant difference at these selected concentrations and times, indicating that RSV had no inhibitory effect on normal cells.

### 2.2. Resveratrol Induces Suffering in OS Cells

**Figure 2:**
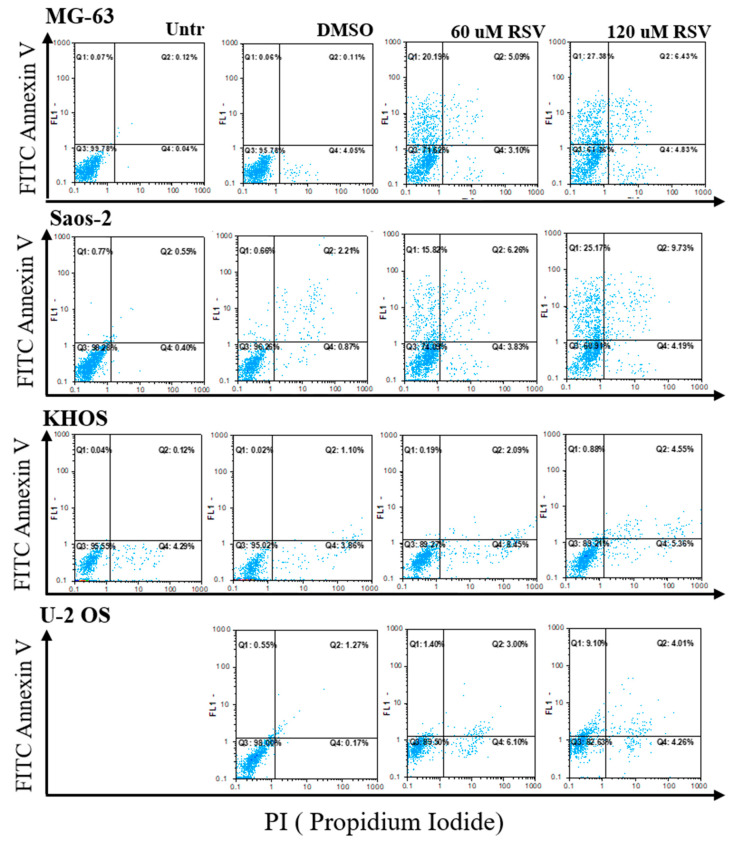
Results of annexin V/PI staining by flow cytometry analysis. For each OS cell line, the results of flow cytometry analysis are reported after 48 h of culture without any treatment (Untreated), or treated with DMSO, or with two different concentrations of RSV (60 or 120 μM). The Q2 quadrant shows the percentage of double-stained cells for annexin V/PI and apoptotic cells. DMSO is the vehicle-related control of RSV and exhibits negligible effect on OS cell lines.

To monitor the OS cell response after RSV treatment, we analyzed annexin V/PI staining by cytofluorimetric analysis. As showed in Figure 2, the OS cells lines after RSV treatment presented an increase in double-staining annexin/PI, demonstrating a slight increase in cell death for apoptosis (Q2).

In addition, global cellular suffering by RSV was highlighted, which included the percentage of single-staining annexin (Q1) and PI (Q4), indicating an increase in preapoptotic (Q1) and necrotic (Q4) cells, respectively. The global suffering percentage is reported in Table 1.

**Figure 3:**
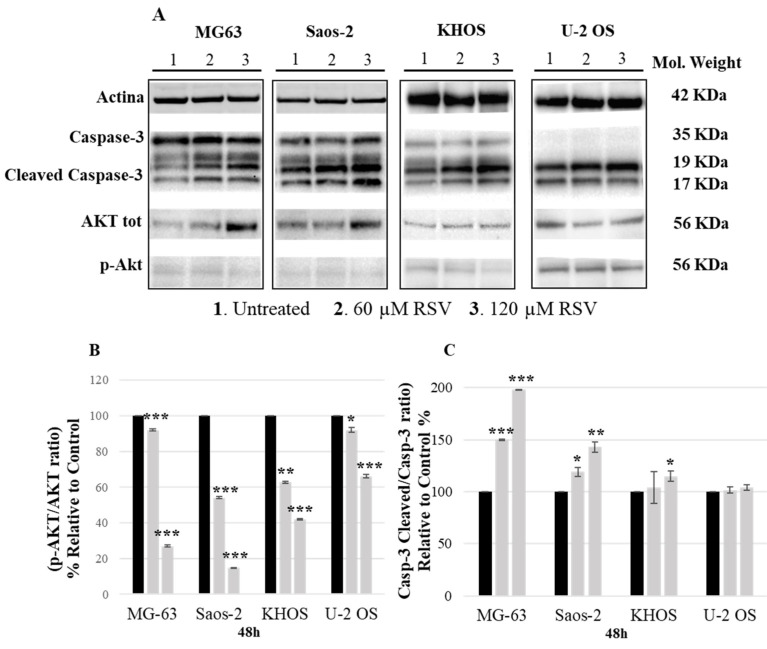
RSV regulates caspese-3 and AKT in OS cells. (**A**)—Representative Western blotting analysis of OS cell expression proteins treated with 60 or 120 μM RSV for 48 h. Actin was used as a load control. (**B**,**C**)—Densitometric quantification of the percentage expression of the ratio p-Akt/Akt (**B**) and cleaved caspase-3/caspase-3 (**C**) of the untreated cells compared to the cells treated with RSV (mean ± SD, *n* = 4). A One-way ANOVA test was used to evaluate the effect of the RSV treatment factor (60 and 120 µM) on the regulation of caspase-3 (MG-63—F = 534, *p* < 0.0005; Saos-2—F = 123, *p* < 0.0005; KHOS—F = 2.5, *p* < 0.0005; U-2 OS—F = 2.2, *p* < 0.0005) and AKT (MG-63—F = 3011, *p* < 0.0005; Saos-2—F = 695, *p* < 0.0005; KHOS—F = 135, *p* < 0.0005; U-2 OS—F = 110, *p* < 0.0005). Dunnett’s test: *, *p* < 0.05; **, *p* < 0.005; ***, *p* < 0.0005 versus untreated cell culture.

To further understand the mechanism underlying the effects of RSV, Western blotting was performed to determine the protein expression levels of *p*AKT and cleaved caspase-3. As shown in Figure 3, we observed a significant reduction in *p*AKT and an increase in cleaved caspase-3 levels in a dose-dependent manner after RSV treatment (Figure 3B,C).

These results indicate that RSV is involved in the *p*AKT and caspase-3 pathway, causing the effects of the inhibition of proliferation and the increase in the percentage of apoptosis, as shown by cytofluorimetric analysis.

### 2.3. Resveratrol Induces an Increase of Expression Levels of the Differentiation Osteoblast Genes and Reduces the Cell Invasion Capabilities

**Figure 4:**
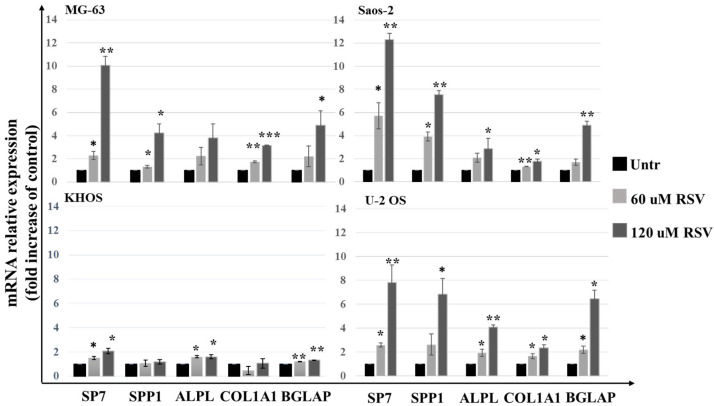
Evaluation of OS cell lines differentiation after RSV treatment in 48 h. qPCR analysis was performed to study the mRNA expression levels of osteoblast differentiation genes. Each bar graph shows the increase in the expression expressed in FOI compared to the control (Untreated). Gene expression analysis was performed using the 2^−ΔΔCT^ method using β-actin expression as the reference gene (mean ± SD, *n* = 4). A one-way ANOVA test was used to evaluate the effect of the RSV treatment factor (60 or 120 µM) on OS cell line differentiation. Dunnett’s test: *, *p* < 0.05; **, *p* < 0.005; ***, *p* < 0.0005 versus untreated cell culture.

Through the qPCR analysis, we evaluated the effects of RSV on the mRNA level expression of osteoblast differentiation genes. In Figure 4, we observed a significant increase in transcription factor Sp7, also called osterix (Osx), which upregulates downstream the expression mRNA levels of genes involved in the osteoblast differentiation process, such as: osteopontin (SPP1), alkaline phosphatase (ALPL), collagen type I alpha 1 (COL1A1), and osteocalcin (BGLAP) [23].

**Figure 5:**
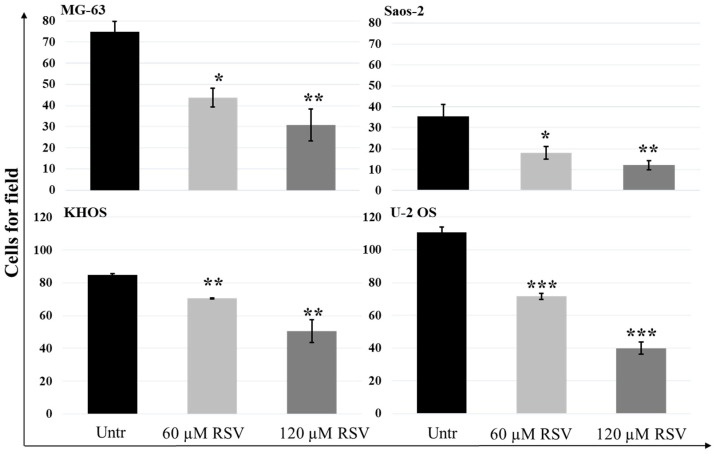
Quantitation of RSV effects on OS cell lines motility by Transwell assay. The number of OS cells that invaded the membrane of the 8 µm Matrigel-coated pores decreased when the cells were treated with an increasing concentration of RSV. The bar charts represent the migration inhibition of OS cell lines as a decreasing number of cells counted in each field. For each cell culture, 5 fields were randomly selected, and the cell number was counted under a light microscope (mean ± SD, *n* = 4). A one-way ANOVA test was used to evaluate the effect of RSV treatment factor (60 and 120 µM) on OS cell line migration (MG-63—F = 23.3, *p* < 0.0005; Saos-2—F = 36.9, *p* < 0.0005; KHOS—F = 75.5, *p* < 0.0005; U-2 OS—F = 564, *p* < 0.0005). Dunnett’s test: *, *p* < 0.05; **, *p* < 0.005; ***, *p* < 0.0005 versus untreated cell culture.

These data were confirmed by a dose-dependent increase in alizarin red staining, as shown in the microscope light images (Figure S2A). On one hand, we observed an increase of expression in osteoblast differentiation genes with a consequent increase of alizarin red staining in differentiated cells; on the other hand, we found a reduction of motility of OS cell lines (Figure S2B). As reported in Figure 5, the results of the Transwell assay, related to the effects of RSV on the motility of OS cell lines, showed how significantly each OS cell line reduced its motility after treatment in a dose-dependent manner (*p*-value interval, [0.005, 0.05])

### 2.4. Resveratrol Treatments Reduce IL6 and IL8 Secretion Levels in OS Cell Lines by Increasing Global Methylation at Different Sites of Each Promoter

The effects of RSV on the inhibition of proliferation and reduction of motility are also related to the reduction of the secretion of proinflammatory interleukins IL-6 and IL-8, respectively.

**Figure 6:**
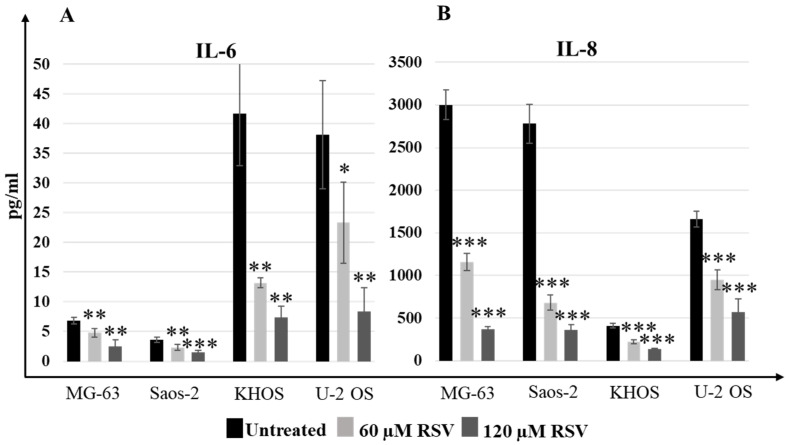
Treatment with RSV reduced IL-6 and IL-8 secretion levels in OS cell lines. The ELISA assay results showed how IL-6 (**A**) and IL-8 (**B**) levels (pg/mL) decreased after 48-h RSV treatment (mean ± SD). Baseline expression of this proinflammatory cytokine was different in each OS cell line, but the decrease in IL-6 levels was observed for each OS cell line. A one-way ANOVA test was used to evaluate the effect of RSV treatment factor (60 and 120 µM) on IL-6 release (MG-63—F = 27.4, *p* < 0.0005; Saos-2—F = 34.7, *p* < 0.0005; KHOS—F= 53.6, *p* < 0.0005; U-2 OS—F = 24.3, *p* < 0.0005) and on IL-8 (MG-63—F = 583, *p* < 0.0005; Saos-2—F = 315, *p* < 0.0005; KHOS—F = 155, *p* < 0.0005; U-2 OS—F = 102, *p* < 0.0005). Dunnett’s test: *, *p* < 0.05, **, *p* < 0.005, ***, *p* < 0.0005 versus untreated cell culture.

Although the differences in the basal secretion levels of both interleukin (IL-6 and IL-8) were evident in each OS cell line (untreated condition), the ELISA results reported in Figure 6 showed that the reduction in secretion levels of IL-6 and IL-8 in OS cell lines was statistically significant (*p*-value interval, [0.0005, 0.05]) with RSV.

**Figure 7:**
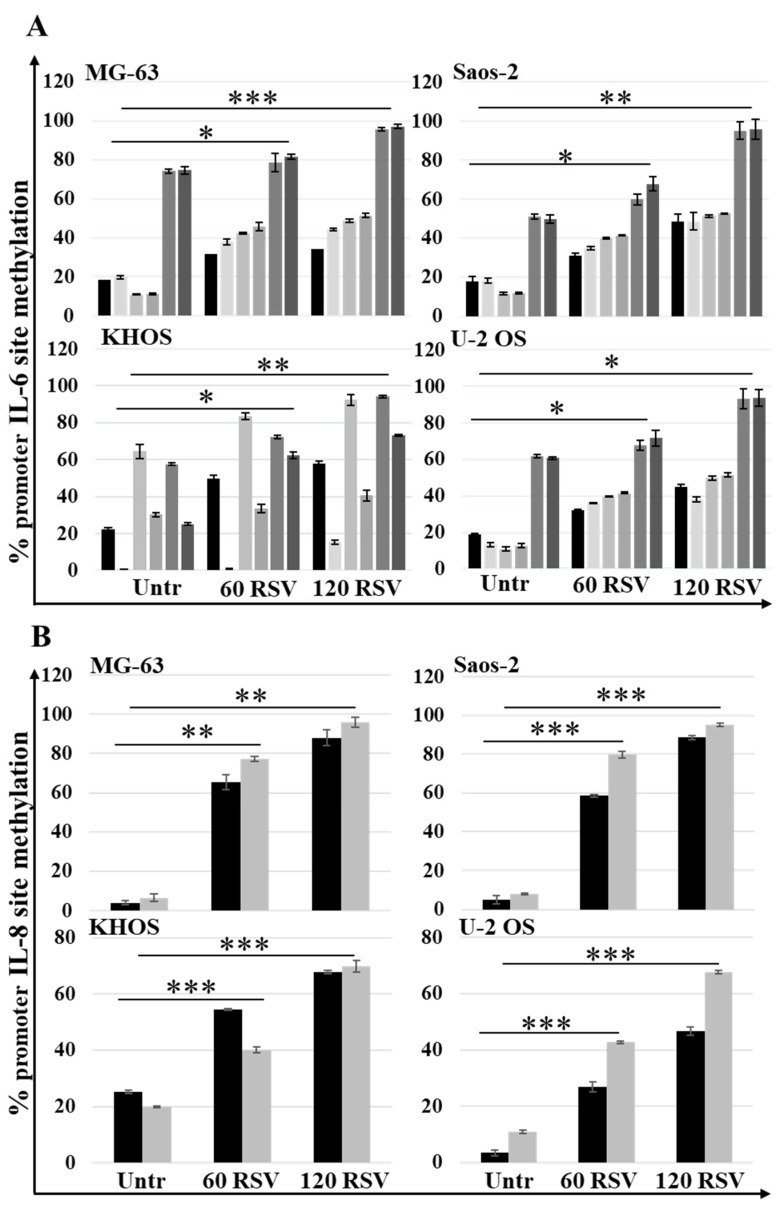
Treatment with RSV induced IL-6 and IL-8 promoter methylation in OS cell lines. MSRE PCR analysis results show the methylation levels of six restriction sites analyzed in 3 regions of the IL-6 promoter (**A**) and the methylation levels of two restriction sites analyzed in 2 regions of the IL-8 promoter (**B**). Data are reported as mean ± SD of *n* = 4 triplicates. A one-way ANOVA test was used to evaluate the effect of the RSV treatment factor (60 and 120 µM) on restriction sites methylation in OS cell lines. Dunnett’s test: *, *p* < 0.05, **, *p* < 0.005, ***, *p* < 0.0005 versus untreated cell culture.

Analyzing the methylation status in six sites of IL-6 promotor and in two sites in IL-8 promotor, assessed by MSRE-PCR, both in untreated and RSV-treated cells, a significant increase (*p*-value interval, [0.0005, 0.05]) in the methylation rate of these sites was found, as reported in Figure 7A,B. This increase in methylation status, observed in each promoter, was related to the reduction in the secretion levels of the proinflammatory interleukins IL-6 and IL-8 since it makes the promoter less accessible to transcription factors.

### 2.5. Therapeutic Effects of Resveratrol and Chemotherapeutic Agent Combination Treatment on OS Cell Line Cultures

**Figure 8:**
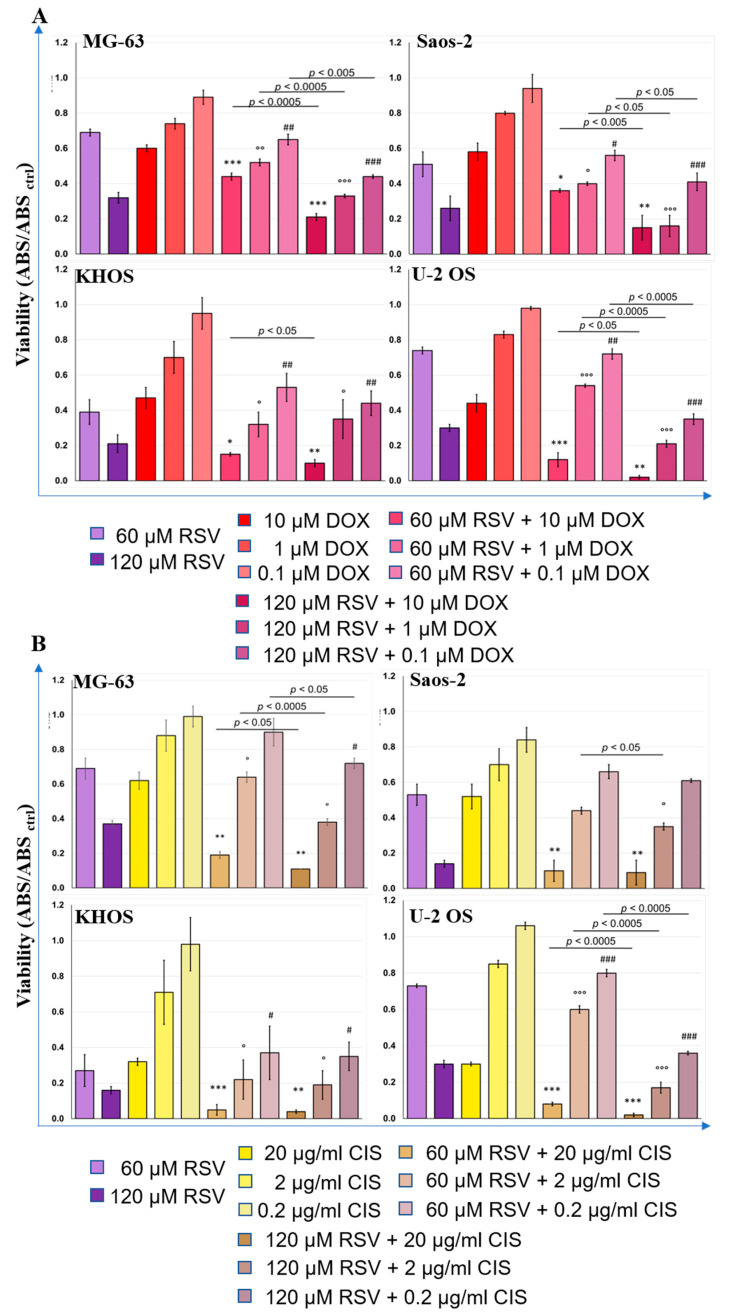
Results of RSV–DOX (**A**) and RSV–CIS (**B**) effects on OS cell lines viability (WST-1) (mean ± SD, *n* = 4). Each OS cell line culture was RSV pretreated (60 or 120 µM) in 48 h and then treated with DOX (10, 1, or 0.1 µM) or CIS (20, 2, or 0.2 µg/mL) within 24 h, except for control treatment cultures. These last were treated with DOX (10, 1, or 0.1 µM) or CIS (20, 2, or 0.2 µg/mL) within 24 h or with RSV (60 or 120 µM) within 48 h. The WST-1 values of OS cell control cultures (ABS_CTRL_) correspond to 1.0. After having observed a significant effect of the combined treatment factor (RSV–DOX or RSV–CIS) on cell viability ONE, pairwise comparison tests between treatment combinations and relative treatment controls were done. For each symbol (*, IC_50_, °, 10^−1^ IC_50_ and #, 10^−2^ IC_50_ dose of DOX or CIS) 1 symbol corresponds to *p* < 0.05; 2 symbols, *p* < 0.005; and 3 symbols, *p* < 0.0005. The line over bars highlights the presence of a significant difference between the two bars.

The inhibitory effects on cellular proliferation of each chemotherapeutic agent, DOX and CIS, was tested by WST-1 assay to calculate the IC_50_ for each chemotherapeutic agent (Figure S3). The IC_50_ concentration of each drug was defined to treat OS cells after their pretreatment with RSV, always at 48 h. Figure 8 shows the WST-1 test results for each cell line and for the combinations RSV–DOX (Figure 8A) and RSV–CIS (Figure 8B).

The chemotherapeutic agents were administered in three concentrations, starting from their IC_50_ and diluting this concentration to 10^−1^ and 10^−2^. We observed a significant reduction in tumor cell proliferation after RSV–DOX or RSV–CIS cotreatments compared to the results achieved with RSV or chemotherapeutic agents alone. In particular, it was highlighted that an important decrease in cell proliferation was already evident at the lowest RSV–DOX and RSV–CIS concentration in comparison to the chemotherapeutic drug. Regarding the comparisons between the different RSV–DOX and RSV–CIS cotreatments with the IC_50_ dose of the chemotherapeutic agents administered (DOX: 10 µM; CIS: 20 µg/mL); Table S1 reports the effect sizes (*d*-value) and relative *p*-values of each comparison. The most significant effects occurred in the cotreatments with the chemotherapy dose corresponding to the IC_50_. For the RSV-DOX cotreatment, the *d*-values of the tested OS cell lines ranged from −7.2 to −3.0 when the RSV dose of 60 µM was administered and from −6.3 to −1.2 when RSV dose was equal to 120 µM. A similar situation occurred for the RSV-CIS cotreatment ranging from −9.0 to −2.9 with 60 µM RSV and from −10.6 to −1.3 for that of 120 µM. The *d*-values indicated that the presence of RSV in the cotreatment with DOX or CIS at the IC_50_ dose enhanced their effects in reducing cell viability. The decrease in WST-1 for the RSV_60µM_–DOX and RSV_120µM_–DOX was about 26–72% and 15–95%, respectively, in comparison with the DOX_IC50_ dose_._ As well as the decrease in WST-1 for the RSV_60µM_–CIS and RSV_120µM_–CIS was about 69–84% and 82–93%, respectively, in comparison with the CIS_IC50_ dose.

With the concentration of the 10^−1^ IC_50_ dose of chemotherapeutic agents always associated with RSV, significant effects in the decrease in cell viability were still observed, even if they were slightly lower than those of cotreatments with the IC_50_ dose of DOX and CIS (Table S1). These variations of WST1 results were present in all OS cell lines in the cotreatments with 120 µM RSV, decreasing by 25–72% for RSV_120µM_–DOX and by 32–82% for RSV_120µM_–CIS, while with 60 µM RSV, the decrease in cell viability was 13–32% for RSV_60µM_–DOX and 15–31% for RSV_60µM_–CIS, except for the U-2 OS line that showed an increase in viability when compared to the cultures treated with IC_50_ DOX and CIS.

With the further reduction to the 10^−2^ IC_50_ dose of chemotherapeutics, always in association with RSV, fewer significant effects were observed regarding a possible further reduction in cell viability compared to the IC_50_ alone, which were observed instead with the RSV_120µM_–DOX with 120 µM RSV (Table S1 and Figure 8).

## 3. Discussion

OS is the most common primary bone sarcoma, and, to date, it continues to affect the lives of too many children, adolescents, and young adults, recurrently manifesting within the metaphysis of long-growing tubular bones [24]. The behavior of OS is like other types of solid tumors so the primary management of OS becomes critically important to ensure success in the majority of patients [25]. Nowadays, the results obtained from numerous clinical studies have defined a standard treatment of OS that involves a three-drug chemotherapy regimen for patients under the age of 40, which sees the use of high doses of methotrexate, DOX, and CIS, followed by surgical resection of the lesions, determining the overall survival level of 65–70% at 5 years [3]. Unfortunately, still today, several patients develop metastases with an elective site in the lung, causing a high mortality rate [26], pointing out how 20–30% of patients are refractory to these conventional treatments [27].

However, recent clinical trials, which evaluated new drugs or replacement proposals for the chemotherapy regimen, have provided no major improvements over current therapy [28]. This evidence confronts us with a broader reality, namely, that a plateau in improving the survival rate may have been reached with current therapeutic approaches, and, for this reason, it is necessary to look for new agents to be incorporated into the current chemotherapy treatment to improve patient outcomes. The current trend is towards natural compounds [29], non-toxic substances that arouse great interest and have been extensively studied by the scientific world to explore new solutions as adjuvants to chemotherapeutic agents [30]. The chemopreventive and anticancer effects of RSV, the object of this study, have been documented by in vivo and clinical studies in a wide variety of tumor cell types [31,32,33]. Numerous studies have reported the strong effect of RSV on the inhibition of cellular growth [34] in several cancer cells [35,36,37,38,39,40,41,42], while some data have reported this effect on OS cells [43,44], motivating us to investigate it. Indeed, in line with the data reported in the literature, the current results show how the effects of different concentrations of RSV on OS cell lines (MG-63, Saos-2, KHOS, U-2 OS) at different experimental times (24, 48, or 72 and 144 h), have dose- and time-dependent effects on OS cell proliferation (Figure 1). All these cell lines, of different origin, were used to best represent the various typical characteristics of OS, partially confirming the results achieved by Li Y et al., which showed how OS cells responded in a variable manner to RSV treatment [43]. This explains why by calculating the IC_50_ of RSV, different values were obtained for MG-63 and Saos-2 around 120 µM and for KHOS and U-2 OS around 60 µM. For this reason, we decided to use both RSV doses for current experiments. The cytotoxic effect of RSV is often attributed to the activation of apoptosis through several pathways in different tumor models [33]. After treatment of the OS cell lines, we observed how RSV induced the proapoptotic effect by increasing the cleavage of caspase-3, which was reflected in an increase of the percentage of doubly positive cells for annexin V/PI by FACS analysis, confirming the data reported by Lihua Peng et al. and Li Y et al. [7,43]. FACS analysis showed an overall suffering state of the OS cell lines after treatment, with a percentage of positive cells in apoptosis, preapoptosis, and necrosis, confirming that RSV can activate other pathways to exert the inhibition of growth on OS cell lines. Recently, Xiao et al. demonstrated that inhibitory effect of RSV on U-2 OS cells proliferation is due to the over-expression of miR-139-5p that downregulates Notch-1 [45]. Several inhibitors of Notch signaling, studied for cancer treatment, also induce the inhibition of the AKT pathway [46]; current data show the inhibition of the AKT pathway after RSV treatment through the reduction of *p*AKT levels, confirming indirectly the data obtained by Xiao et al.

Other studies have reported that RSV induces osteoblastic differentiation in pathological and non-pathological conditions [47,48,49]. The treatment with RSV in multiple myeloma cells inhibits the growth of tumor cells and also acts on the microenvironment, inhibiting osteoclastogenesis and, thus, bone resorption and inducing osteoblastic differentiation [50,51]. Analyzing the expression levels of osteoblastic differentiation genes on OS cells lines treated with RSV, we highlight a significant increase in the expression levels of SP7 (Osterix) (Figure 4), a master gene that regulates the mRNA expression levels of the genes downstream involved in the osteoblast differentiation process. These results were validated by the partial increase in the alizarin red staining compared to the untreated cultures, which highlights the calcium deposits in the cell cultures treated with RSV. It performs an action like the inducer of differentiation, which is currently studied to make OS cells more vulnerable to the action of chemotherapeutic agents [52]. Another contribution to tumor growth is due to proinflammatory cytokines, whose role in tumor progression is a well-described process in several cancer models [53]. In particular, it is known that the relevant release of IL-6 and IL-8 by tumor cells has an autocrine effect, promoting tumor growth, angiogenesis, tumor cell motility [54], and the chemo-resistance phenomena [55], which correlate with poor prognosis in many cancers. Monitoring the IL-6 and IL-8 secretion levels from OS cell lines by ELISA assay (Figure 6), a significant decrease of secretion of these proinflammatory cytokines after treatment with RSV was highlighted. This reduction is related to the methylation status of both promotors. Indeed, MSRE-PCR analysis allowed us to compare the methylation status of six sites in the IL-6 promoter and two sites in the IL-8 promoter of RSV-treated cells versus untreated ones, showing an increase in the methylation rate of the specific sites analyzed in each promoter, as reported in Figure 7. The relationship between the increase of the methylation status and the reduction of the secretion levels of IL-6 and IL-8 indicates an epigenetic action of RSV on these genes, explaining further the inhibitory effects of RSV on OS cellular growth and, on the other side, on the inhibitory effects reported on the cellular motility of OS cell lines evaluated by Transwell assay. The inhibitory effect of RSV on tumor cell migration has already been demonstrated on several cancer models such as gastric [56], lung [57], prostate [58], and breast [59]. As reported by Shun-Fa Yang et al., RSV exerts effects on the cell motility of OS cells by the transcriptional and epigenetic regulation of MMP2, inhibiting CREB-DNA-binding activity and upregulating miR-328 [26]. Current investigations confirm the epigenetic effects of RSV on OS cell lines, providing additional information on the methylation status of IL-6 and IL-8 promoters, which contributes to influencing OS cell proliferation and motility, and further demonstrating how RSV acts epigenetically, upstream of MMP2 regulation, increasing the methylation levels of the IL-8 promoter. As reported by Qiaoshi Xu et al. in neck squamous cells, IL-8 over-expression promotes the enhancement of the expression levels of MMP2 and MMP9 [60].

These data represent a strong motivation to explore the combined effects of RSV with chemotherapeutic agents. As reported in the literature, strong evidence on breast, gastric, and prostate cancer cells, subjected to combined treatment with RSV–DOX or RSV–CIS, has shown a synergistic behavior of RSV towards chemotherapeutic agents [61,62,63]. The combined treatments showed variable effects: viability inhibition, apoptosis promotion, epithelial–mesenchymal transition inhibition, intracellular drugs accumulation, and reduction of bone loss due to chemotherapy [17,64,65]. These results have prompted us to investigate the effects of RSV in cotreatment with current chemotherapeutics used in the therapeutic regimen of OS. Indeed, the growth inhibition of OS cell lines exposed to single treatments with different concentrations of DOX and CIS for 24 h, after pretreatment with RSV for 48 h, was evaluated. As reported in Figure 8, these preliminary data showed a significant reduction in cell growth, even when using a concentration ten times lower than the IC_50_ concentration for each drug. 

Current data showed that RSV exerts multiple effects on different processes such as growth inhibition, apoptosis, migration, differentiation, cytokine secretion, and epigenetic effects, supporting its role as an adjuvant agent. These results offer a basis for further peculiar investigations on each of these effects, on the one hand, by analyzing in depth the molecular mechanisms that regulate them and, on the other hand, possible correlations with other pathways activated by RSV, such as the Wnt/β-catenin [66] or ERK-p53 [67] signaling pathway that converges with the reported data in the inhibition of proliferation, increase in apoptosis, inhibition of migration, and invasion capacity of OS cells.

The confirmation of these data in a three-dimensional set-up is an objective to be achieved in order to support the hypothesis that RSV might be tested as an adjuvant in specific clinical protocols.

## 4. Materials and Methods

### 4.1. Reagents

Dulbecco’s modified Eagle’s high glucose medium (DMEM), alpha modified Eagle’s medium (α-MEM), mesenchymal stem cell basal medium (MSCBM), mesenchymal stem cell growth medium single quots supplements and growth factors (MSCGM Single Quots); osteoblast growth and differentiation basal medium (OBM), and osteoblast growth medium single quots supplements and growth factor (OGM Single Quots) were purchased from Lonza Group (Basel, Switzerland); fetal bovine serum (FBS), L-glutamine, and penicillin-streptomycin were purchased from Lonza Group. RSV, ≥99% (HPLC) derived from plant root was purchased from Sigma-Aldrich (R5010, Merck KGaA group, Darmstadt, Germany); 0.9% NaCl and dimethyl sulfoxide (DMSO) were purchased from Sigma-Aldrich (Merck KGaA group, Darmstadt, Germany).

### 4.2. Cell Culture

Human OS cell lines, MG-63, Saos-2, KHOS, and U-2 OS, were purchased from ATCC LGC Standards S.r.L. (Sesto San Giovanni, Milan, Italy). Saos-2 and U-2 OS cells were cultured in DMEM medium supplemented with 10% FBS, and MG-63 and KHOS cells were cultured in α-MEM medium supplemented with 15% FBS. Respectively, in both culture mediums, 1 mM L-glutamine and 100× pen/strep (100 units of potassium penicillin and 100 µg of streptomycin sulfate for 1 mL of culture media) were added. 

Human mesenchymal stem cells (hMSC) and NHOst–human osteoblasts were purchased from Lonza Group (Basel, Switzerland) and used as control cell lines. The hMSC cells were cultured in MSCBM supplemented with the MSCGM Single Quots Kit, while the NHOst cells were cultured in OBM supplemented with the OGM Single Quots Kit. 

The cell cultures were maintained at 37 °C in a humidified atmosphere containing 5% CO_2_.

### 4.3. Cell Cultures Treatment Protocols

Cultured cells were treated with several concentrations of RSV in complete medium for each cell line.

RSV treatments: OS cell lines were treated with a range of RSV concentrations (0–10–25–100 µM) at several experimental times points to define the IC_50_ (AAT Bioquest, Inc. (1 February 2022, Sunnyvale, CA, USA); Quest Graph™ IC_50_ Calculator; https://www.aatbio.com/tools/ic50-calculator accessed on 14 February 2022). Experiments were performed using untreated OS cell lines as control, and DMSO solution (DMSO) was dissolved in complete medium at a final concentration of 120 µM as vehicle control because the RSV was dissolved in DMSO. After having defined the IC_50_ value of RSV in each OS cell line, each experiment was performed by using 60 and 120 µM RSV in 48 h. This experimental time was chosen as we observed a significant effect of the RSV compound on OS cell lines.

**Figure 9:**
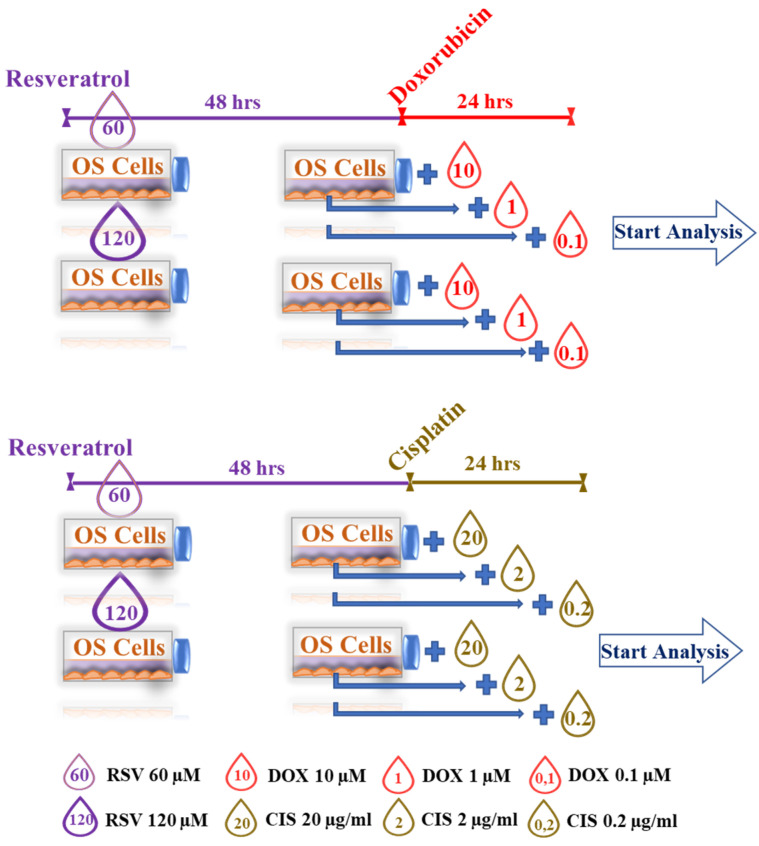
Set-up of RSV–DOX and RSV–CIS cotreatments.

RSV–DOX and RSV–CIS cotreatments: They were set to evaluate the combined effect of pretreatment of OS cell lines with doses of IC_50_ RSV (60 and 120 µM) for 48 h, with subsequent administration of DOX or CIS at different concentrations for 24 and 48 h. The concentrations of DOX and CIS were calculated, starting from the respective doses of IC_50_ and decreasing these concentrations to 10^−1^ and 10^−2^ (Figure 9).

Within these experiments, we used different controls for each OS cell line: untreated cultures (UNTR), cultures treated with DMSO, or 0.9% NaCl as vehicle controls since RSV and DOX must be dissolved in DMSO and CIS in 0.9% NaCl.

### 4.4. Cell Proliferation Assay

The WST-1 assay was used to evaluate the cellular growth (Roche Diagnostics GmbH, Mannheim, Germany). The assay was performed in a 96-well plate according to the recommendations of the manufacturer; 2.5 × 10^3^ cells were seeded per well in 96-well flat-bottomed plates in 100 µL cell culture medium specific for each cell line and incubated for 24 h.

The cells were then treated accordingly to the defined treatment protocols, as reported in Figure 9. The medium was changed after three days, after the cells were cultured for six days. Cell growth was analyzed at 24, 48, 72, and 144 h of culture. Before measurement, a 1/10 volume of WST-1 reagent was added to each well, and the cells were incubated at 37 °C for 3 h. The absorbance of the samples was measured against the background (culture medium plus WST-1 reagent w/o cells) at 450/650 nm. The absorbance values obtained from the WST-1 assay are proportional to the total number of living cells.

### 4.5. Annexin V/PI Double Staining

OS cells (2.5 × 10^6^ seeded in 6-well plates) were treated for 48 h with two different concentrations of RSV (60 and 120 µM). Then, the cells were trypsinized and stained with conjugated annexin V–FITC and propidium iodide (PI) using the FITC Annexin V Apoptosis Detection Kit 1 (Becton Dickinson Italia S.p.A., Milan, Italy), according to the manufacturer’s instructions. The stained cells were immediately analyzed by a Partec CyFlow Space Flow Cytometer (SYSMEX PARTEC GMBH, Goerlitz, Germany).

### 4.6. Quantitative PCR (qPCR) Analysis

Total RNA was extracted from the scaffold using Trizol reagent (Invitrogen™, Waltham, MA, USA). Each cDNA sample was tested in triplicate. Quantitative RT-PCR analysis was performed in LineGene 9640 Bioer (CaRli biotec S.r.l, Roma, Italy) using SYBR^®^ Green Real-Time PCR Master Mixes (Applied Bio-systems™, Life Technologies—EuroClone S.p.A, Pero, Milan, Italy). QuantiTect Primers (Qiagen Srl, Milan, Italy) and designed primers (Invitrogen™) were used (Table S2). The expression of target genes was analyzed performing the 2^−ΔΔCT^ method, where the β-actin expression was used as the reference gene. The results obtained are expressed as the “relative fold” of changes calculated with respect to untreated cells; these data were used as a calibrator for each experimental point. All procedures have been performed in accordance with the manufacturer’s instructions.

### 4.7. MSRE PCR Analysis

The isolation of genomic DNA was carried out with the PureLink Genomic DNA mini-Kit (Invitrogen™, Waltham, MA, USA). The obtained DNA was quantified using A Nanodrop 2000 spectrophotometer (ThermoFisher Scientific, Waltham, MA, USA) and successively analyzed in 0.6% agarose gel electrophoresis to control the integrity of DNAs. Methylation sensitive restriction endonuclease–PCR (MSRE–PCR) analysis [68] was performed in order to determine the methylation status of the CpG-rich sites, present in the 5′ flanking region of the proximal promotor of interleukin-6 (IL-6) and interleukin-8 (IL-8). Essentially, genomic DNA was digested with HpaII or HhaI, methylation-sensitive restriction endonucleases (MSREs), and then amplified by PCR in the presence of primers flanking the three regions of IL-6 (all PCR products containing one cutting site for HpaII and one cutting site for HhaI) and two regions of the proximal promotor of IL-8 (containing one of the cutting sites for HpaII and the other containing one cutting site for HhaI). PCR reaction mixtures containing 200 ng of DNA (treated with restriction endonuclease or not), 0.2 µM of specific primers, 0.2 mM of deossiribonucleotides triphosphates, and 2.5 U Taq polymerase (Invitrogen™) were amplified using the following protocol: initial denaturation step at 94 °C for 4 min, followed by 30 cycles of 94 °C for 1 min (denaturation step), 65 °C for 1 min (annealing step), and 72 °C for 1 min (elongation step), and, finally, a terminal extension step at 72 °C for 5 min [69]. PCR products were analyzed by 2% agarose gel electrophoresis, visualized by Gel Red staining (Biotium, Hayward, CA, USA) in a ChemiDoc apparatus (Bio-Rad Laboratories, Hercules, CA, USA), the image captured in the digital support and densitometric analysis obtained using the “Image Lab” application (version 5.2.1) of Bio-Rad Laboratories.

### 4.8. Western Blot Analysis

SDS-PAGE electrophoresis and Western blotting were performed using cells lysed for 1 h in NP40 Cell lysis buffer containing 50 mM Tris, pH 7.4, 250 mM NaCl, 5 mM EDTA, 50 mM NaF, 1 mM Na3VO4, 1% Nonidet P40 (NP40), and 0.02% NaN3 (Invitrogen™); 1 mM PMSF (1M, Sigma–Aldrich) and Protease Inhibitor Cocktail (100X, Sigma–Aldrich) were added to the cell lysis buffer. To separate the cell lysates (30 µg per lane), we used the 4–12% Novex Bis-Tris SDS-acrylamide gels (Invitrogen™), transferred on Nitrocellulose membranes (Invitrogen™), and immunoblotted with the primary antibodies. The following antibodies against the following proteins were used: β-actin (sc-47778), caspase-3 (8G10) (1:1000, #9665—Cell Signaling Technology, Inc., Danvers, MA, USA), cleaved caspase-3 (Asp175)—(5A1E) (1:1000, #9664—Cell Signaling), AKT (B1) (1:200, sc-5298), p-Akt1 (5.Ser 473) (1:200, sc-293125), and the secondary anti-mouse IgG HRP-linked antibody (1:2000, #7076—Cell Signaling) and anti-rabbit IgG HRP-linked antibody (1:2000, #7074—Cell Signaling).

### 4.9. Enzyme-Linked Immunosorbent Assay

To detect the secretion of IL-6 and IL-8 in the supernatant, 1.5 × 10^4^ cells were plated in 12-well plates. The cells were then treated with and without RSV for 48 h. After centrifugation at 1200 rpm for 5 min, the supernatant was collected to measure IL-6 and IL-8 levels by an enzyme-linked immunosorbent assay (ELISA) kit (R&D Systems Europe, Ltd., Abingdon Science Park, Abingdon, UK) according to the manufacturer’s instructions.

### 4.10. In Vitro Cell Migration Assay

To perform the migration assay, the OS cells were treated for 24 h before with two concentrations of RSV (60 and 120 µM) to observe the effects of RSV on migration capabilities of the cells through the filter. The assay was performed using Cell Culture Inserts (8 µm pore size; Corning Life Sciences, Union City, CA, USA); they were placed into a 24-well cell culture plate, and the upper compartments were coated with 50 µL with Matrigel (1 g/L) at 37 °C for 1 h. The cells were suspended at a final concentration of 2 × 10^5^ cells/mL in 500 µL serum-free medium and seeded into the upper chamber, while the lower chamber was filled with 500 µL complete culture medium for each cell line. After 24 h in culture, each permeable filter of the insert was fixed using 4% paraformaldehyde, and the cells stratified on the upper surface were scraped off. The cells that had migrated through the membrane pores of the insert and were distributed at the lower surface were stained with 0.1% crystal violet and counted under a microscope. Five fields were randomly selected, and the cell number was counted under a light microscope (Nikon Eclipse TI-S, Nikon Europe BV, Amsterdam, The Netherlands) [70].

### 4.11. Red Alizarin Assay

The red alizarin assay was used to evaluate the calcium deposition in OS cell lines after treatment for 48 h with RSV (60 and 120 µM). The OS cell lines were seeded to 1.5 × 10^4^ cells/well in 12-wells plates, then treated with RSV. After this time, every well was washed with PBS and fixed using 4% PFA at room temperature for 30 min. Double-distilled water (ddH_2_O) was used to rinse the plates twice; the plates were incubated with 2% alizarin red S (ARS) staining solution (Sigma-Aldrich) at room temperature for about 30 min. Finally, cells were gently washed by ddH_2_O, and images were observed via a light microscope (Nikon Eclipse TI-S) [71].

### 4.12. Statistical Analysis

Statistical analysis was performed using R software v.4.1.2 [72]. The normal distribution of data by the Shapiro–Wilk test and their variance homogeneity by the Levene test were verified. ANOVA was used to test if the significant effects of fixed factor—“treatment” or interactions of fixed factors—“treatment” and “experimental time”, respectively, were present in the cell viability (WST-1), gene expression, protein expression, ELISA, cell migration, and methylation results. Pairwise comparisons of data were carried out, and Sidak’s adjusted *p*-values were calculated; the estimator *d*_msw_ (hereafter reported as *d*) was used to calculate the effect size of significant comparisons [73]. Data are reported as mean ± SD at the significant level of *p* < 0.05.

## 5. Conclusions

The encouraging results obtained and supported by the literature confirm that RSV acts on a broad spectrum on several cellular mechanism-sensitizing OS cells to the action of chemotherapeutic agents. This is a good assumption that motivates us to explore other mechanisms of action of RSV because, in light of these data, the use of RSV could be evaluated as a possible adjuvant of chemotherapeutic agents already used in therapeutic protocols for the pharmacological treatment of OS. In particular, the combined treatment of RSV with chemotherapeutic agents would allow the use of lower doses of DOX and CIS, which could lead to a reduction in the side effects associated with the chemotherapeutic agents and the chemoresistance phenomenon and to the reduction in therapeutic costs for national health systems.

## Figures and Tables

**Table 1:** The percentages reported summarize the suffering state of the OS cell lines due to RSV treatments. The percentage values of viable cells or unstained cells are given by the percentages reported in the Q3 quadrants of each flow cytometry analysis. The percentage values of the suffering cells are given by the sum of the percentage values of the quadrants Q1 (annexinV positive), Q2 (annexin V/PI positive), Q4 (PI positive) for each cell line.

Treatments	MG-63		Saos-2		KHOS		U-2 OS	
	*Viable*	*Suffering*	*Viable*	*Suffering*	*Viable*	*Suffering*	*Viable*	*Suffering*
Untreated	99.8	0.2	98.3	1.7	95.6	4.4	99.8	0.2
DMSO	99.5	4.2	96.3	3.7	95.0	5.0	98.0	2.1
60 µM RSV	71.6	28.4	74.1	25.9	89.3	10.7	89.5	10.5
120 µM RSV	71.3	38.7	60.9	39.1	89.2	10.8	82.6	17.4

## Data Availability

Data is contained within the article and supplementary material.

## Supplementary Materials

The following supporting information can be downloaded at: https://www.mdpi.com/article/10.3390/ph15030342/s1, Figure S1: Effects of resveratrol on the proliferation of not-tumorigenic cells.; Figure S2: Microscopic images of alizarin red staining and Transwell assays of OS cell lines treated with IC_50_ RSV; Figure S3: Effects of chemotherapy agents on OS cell lines evaluated by WST-1 assay after 24 h; Table S1: Effect sizes (*d*-values) of pairwise comparisons of WST-1 results of RSV-DOX and RSV-CIS cotreatments in comparison with IC_50_ DOX and CIS treatments, respectively (*, *p* < 0.05, **, *p* < 0.005, ***, *p* < 0.0005); Table S2: List of gene primers involved in the osteoblast differentiation process.
