# Supplementary material for: Multiple Effects of Resveratrol on Osteosarcoma Cell Lines
Source: Pharmaceuticals (Basel). 2022 Mar 11;15(3):342. doi: 10.3390/ph15030342 (PMC8956103; doi:10.3390/ph15030342)
Supplement: Supplementary file 1 [file pharmaceuticals-15-00342-s001.zip › pharmaceuticals-1619101-supplementary.pdf]

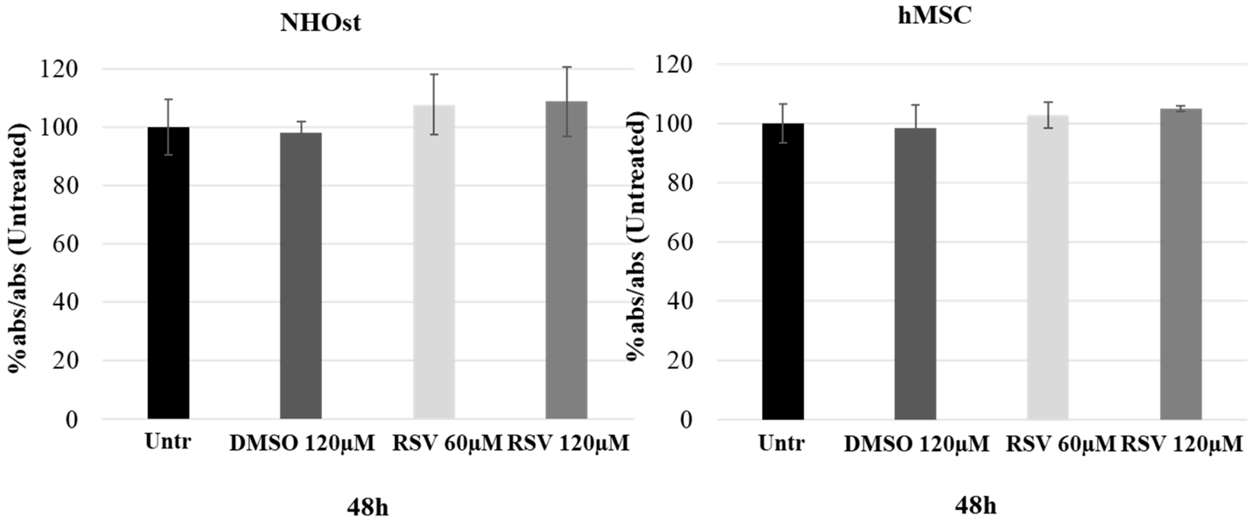

Supplementary Figure S1. Effect of Resveratrol on proliferation of not tumorigenic cells. Cell viability is tested by WST-1 assay at several times after 48h. As reported in each histogram the absorbance values are converted into percentage absorbance values respect to the untreated cells of each human primary cell lines, normal human osteoblasts (NHOst) and human mesenchymal stem cells (hMSC). Two IC50 RSV concentrations estimated on OS cell lines were tested on non-tumor cell cultures. As shown in the graphs, treatment with RSV has no inhibitory effect on proliferation.

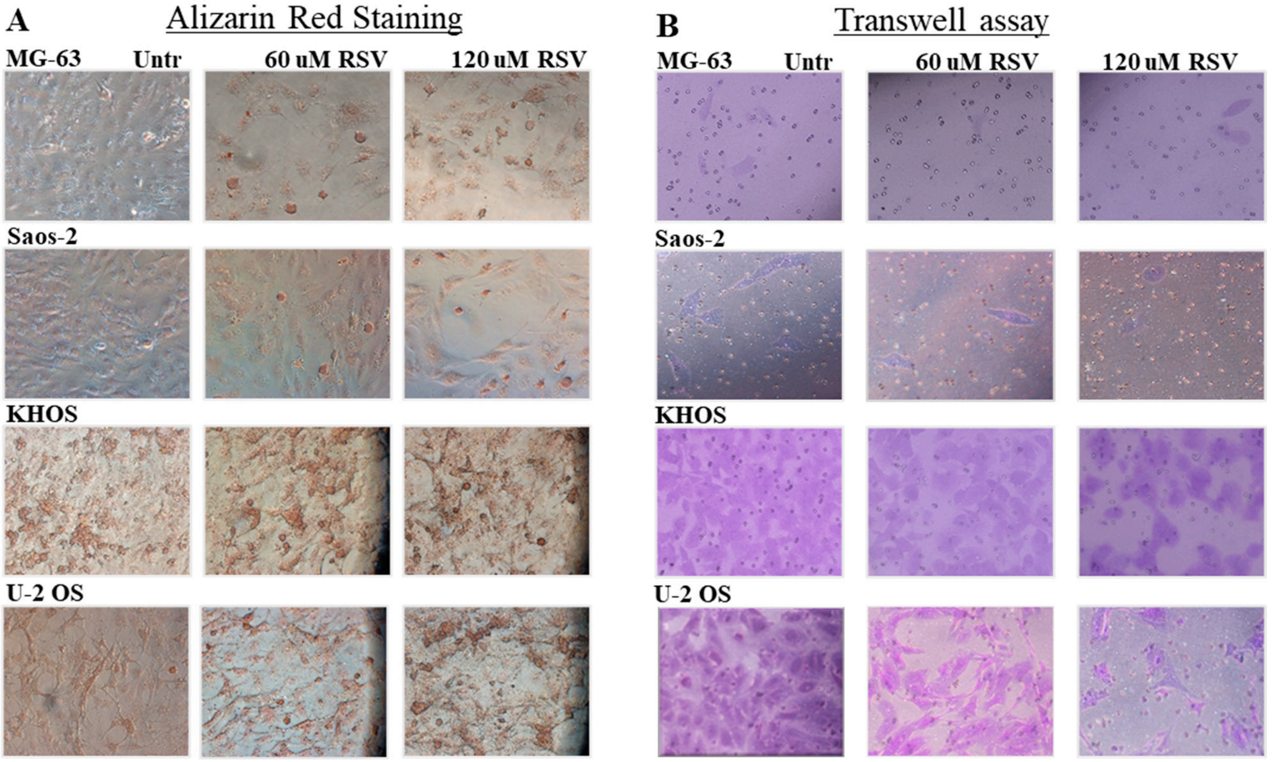

Supplementary Figure S2. (A) - Representative images (magnification: 20x) of OS cell lines after 48-hour treatment with RSV and stained with alizarin red are shown on the right of each histogram. Alizarin red staining is more intense in OS cell lines treated with RSV 60 and 120 µM, compared to untreated cells. (B) - The number of OS cells that invaded the membrane of the 8 µm Matrigel-coated pores decreased when the cells were treated with increasing concentration of RSV (representative micrographs, magnification 20x).

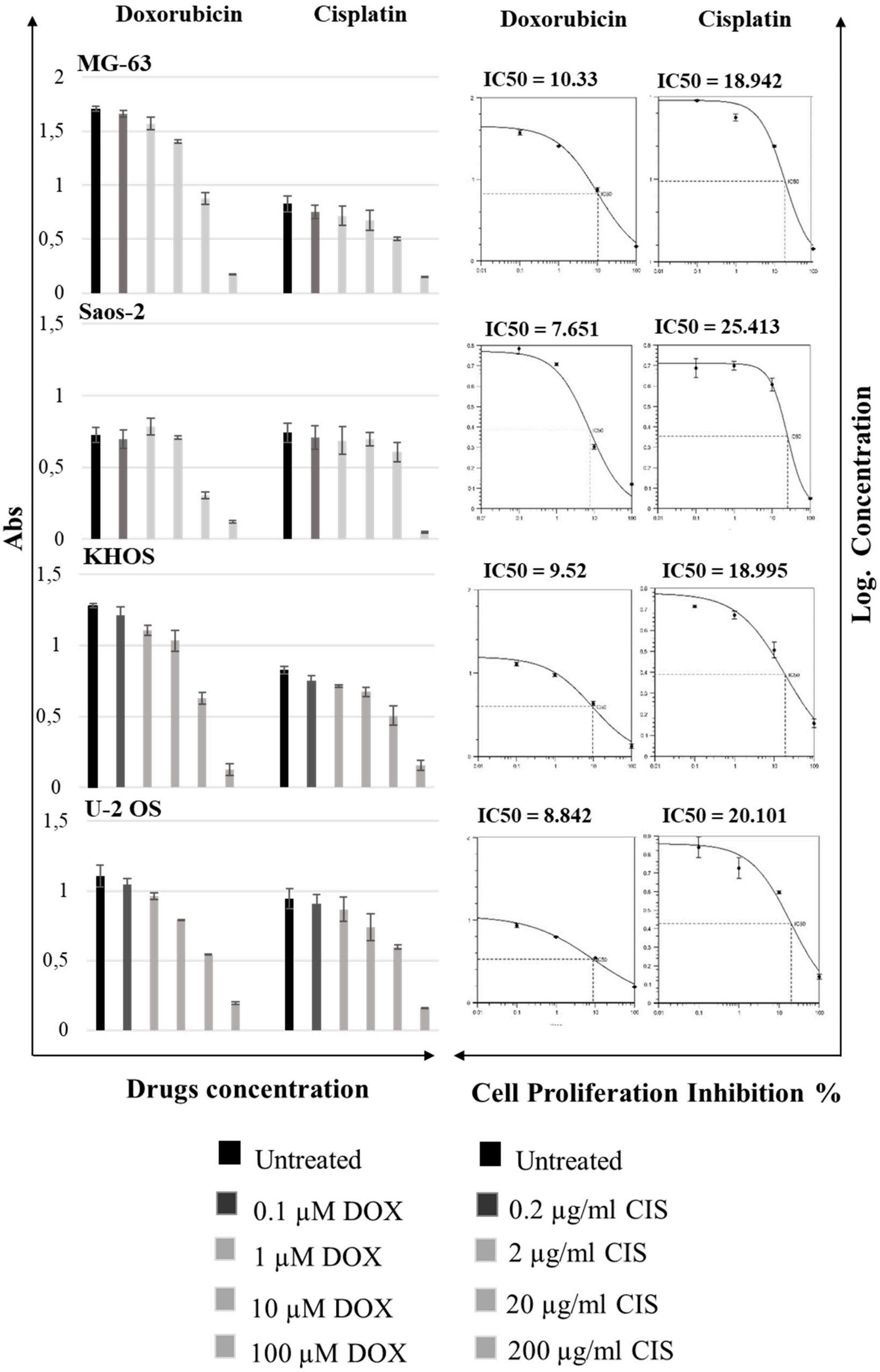

Supplementary Figure S3. Effects of chemotherapy agents on OS cell lines evaluated by WST-1 assay after 24 hours. Preliminarly, OS cell lines were treated with several concentration of DOX (0.1-100 uM) and CIS (0.1-100 ug/ml) to determine the IC_{50} for each drug. Cell viability was reported as absorbance results of WST-1 assay (Mean±SD, n=3). On the right of each histogram are reported the graphs of IC50 values of DOX and CIS calculated for each cell lines respectively, after 24 hours.

Supplementary Table S1. Effect sizes (*d*-value) and relative *p*-values of the comparisons between the different RSV-DOX and RSV-CIS co-treatments with the IC_{50} dose of the chemotherapeutic agents administered (DOX: 10 μM; CIS: 20 μg/ml).

TREATMENTS				MG-63		SAOS-2		KHOS		U2-OS	
RSV +	Drug		Drug IC50	*d*	*p*	*d*	*p*	*d*	*p*	*d*	*p*
60 μM	10μM DOX	vs	10μM DOX	-4.3	***	-3.0	*	-3.5	***	-7.2	***
60 μM	1μM DOX	vs	10μM DOX	-2.1	**	-2.4	*	-1.6	*	2.2	**
60 μM	0.1μM DOX	vs	10μM DOX	1.3	*	-0.3	ns	0.7	ns	6.3	***
120μM	10μM DOX	vs	10μM DOX	-2.9	***	-1.5	**	-1.2	***	-6.3	***
120μM	1μM DOX	vs	10μM DOX	0.3	***	-1.3	*	1.5	*	-2.0	***
120μM	0.1μM DOX	vs	10μM DOX	-3.2	***	-2.0	*	-2.5	ns	-1.1	**
60 μM	20μg/ml CIS	vs	20μg/ml CIS	-9.0	**	-4.6	***	-2.9	***	-8.3	***
60 μM	2μg/ml CIS	vs	20μg/ml CIS	0.4	ns	-0.9	ns	-1.1	*	11.3	***
60 μM	0.2μg/ml CIS	vs	20μg/ml CIS	5.8	**	1.5	*	0.5	ns	18.9	***
120μM	20μg/ml CIS	vs	20μg/ml CIS	-5.4	**	-4.7	**	-1.3	***	-10.6	***
120μM	2μg/ml CIS	vs	20μg/ml CIS	0.2	**	-1.9	*	0.3	**	-4.9	***
120μM	0.2μg/ml CIS	vs	20μg/ml CIS	7.3	*	1.0	ns	2.0	ns	2.3	***

Supplementary Table S2. List of gene primers involved in osteoblast differentiation process.

Gene	Qiagen Primers	Catalog no.	Annealing temperature (°C)
SP7	Hs_SP7_1_SG QuantiTect Primer Assay	QT00213514	60
ALPL	Hs_ALPL_1_SG QuantiTect Primer Assay	QT00012957	60
BGLAP	Hs_BGLAP_1_SG QuantiTect Primer Assay	QT00232771	60
SPP1	Hs_SPP1_1_SG QuantiTect Primer Assay	QT01008798	60
COL1A	Hs_COL1A1_1_SG QuantiTect Primer Assay	QT00037793	60
ACTB	Hs_ACTB_1_SG QuantiTect Primer Assay	QT00095431	60

List of Qiagen gene primers involved in the differentiation osteogenic process. Expression was normalized using the β-ACTIN reference gene (*ACTB*).
